# CD8^+^ T cells induce platelet clearance in the liver via platelet desialylation in immune thrombocytopenia

**DOI:** 10.1038/srep27445

**Published:** 2016-06-20

**Authors:** Jihua Qiu, Xuena Liu, Xiaoqing Li, Xu Zhang, Panpan Han, Hai Zhou, Linlin Shao, Yu Hou, Yanan Min, Zhangyuan Kong, Yawen Wang, Yu Wei, Xinguang Liu, Heyu Ni, Jun Peng, Ming Hou

**Affiliations:** 1 Department of Hematology, Qilu Hospital, Shandong University, Jinan, China; 2 School of Medicine, Zhejiang University, Hangzhou, China; 3 Canadian Blood Services and the Department of Laboratory Medicine and Pathobiology, Keenan Research Centre in the Li Ka Shing Knowledge Institute of St. Michael’s Hospital, University of Toronto, Toronto, Canada; 4 Key Laboratory of Cardiovascular Remodeling and Function Research, Chinese Ministry of Education and Chinese Ministry of Health, Qilu Hospital, Shandong University, Jinan, China; 5 Shandong Provincial Key Laboratory of Immunohematology, Qilu Hospital, Shandong University, Jinan, China

## Abstract

In addition to antiplatelet autoantibodies, CD8^+^ cytotoxic T lymphocytes (CTLs) play an important role in the increased platelet destruction in immune thrombocytopenia (ITP). Recent studies have highlighted that platelet desialylation leads to platelet clearance via hepatocyte asialoglycoprotein receptors (ASGPRs). Whether CD8^+^ T cells induce platelet desialylation in ITP remains unclear. Here, we investigated the cytotoxicity of CD8^+^ T cells towards platelets and platelet desialylation in ITP. We found that the desialylation of fresh platelets was significantly higher in ITP patients with positive cytotoxicity of CD8^+^ T cells than those without cytotoxicity and controls. *In vitro*, CD8^+^ T cells from ITP patients with positive cytotoxicity induced significant platelet desialylation, neuraminidase-1 expression on the platelet surface, and platelet phagocytosis by hepatocytes. To study platelet survival and clearance *in vivo*, CD61 knockout mice were immunized and their CD8^+^ splenocytes were used. Platelets co-cultured with these CD8^+^ splenocytes demonstrated decreased survival in the circulation and increased phagocytosis in the liver. Both neuraminidase inhibitor and ASGPRs competitor significantly improved platelet survival and abrogated platelet clearance caused by CD8^+^ splenocytes. These findings suggest that CD8^+^ T cells induce platelet desialylation and platelet clearance in the liver in ITP, which may be a novel mechanism of ITP.

Primary immune thrombocytopenia (ITP) is an autoimmune disorder characterized by a low platelet count and an increased risk of bleeding. CD8^+^ T cells can directly lyse platelets and play an important role in the increased platelet destruction observed in ITP patients^1^,^2^,^3^,^4^,^5^. Additionally, CD8^+^ T cells may also suppress megakaryocyte apoptosis, leading to impaired platelet production in ITP^6^. However, considering that antigen-specific CD8^+^ cytotoxic T lymphocytes (CTLs) are relatively few, other mechanisms accounting for CD8^+^ T cell-mediated thrombocytopenia likely exist in ITP.

Platelet desialylation is the process in which terminal sialic acids are cleaved from glycoconjugates, mainly glycoproteins (GPs), on the platelet surface^7^,^8^. The loss of sialic acids causes exposure of the penultimate β-galactose residues. The residues are recognized by the asialoglycoprotein receptors (ASGPRs), which are also called Ashwell-Morell receptors, on hepatocytes. Desialylated platelets are captured and phagocytosed by ASGPRs-expressing hepatocytes in the liver^9^,^10^,^11^,^12^,^13^. Over the past decades, platelet desialylation has been shown to be responsible for platelet clearance in many contexts, such as the destruction of chilled platelets^12^,^14^,^15^,^16^,^17^, free radicals and infection-related thrombocytopenia^18^,^19^,^20^,^21^, and the clearance of senescent platelets^22^,^23^. Neuraminidase-1 (Neu1) is a lysosomal neuraminidase that hydrolyzes sialic acids from glycoproteins preferentially^24^,^25^. Jansen *et al*. analysed total human platelet lysates and found that resting platelets contained an internal pool of Neu1, which could translocate to the platelet surface after platelet lesion and catalyze platelet desialylation^17^.

It has been demonstrated that both the liver and spleen are critical sites for platelet clearance in ITP, and that liver sequestration is significantly increased in splenectomized ITP patients^26^. Because desialylated platelets are phagocytosed in the liver^27^,^28^, it is possible that platelet desialylation contributes to the increased platelet destruction in ITP. Both antiplatelet autoantibodies and CD8^+^ T cells are involved in the abnormal immune recognition of autologous platelets in ITP. Recent studies have demonstrated that anti-GPIb autoantibodies induce platelet desialylation and Neu1 expression on the platelet surface, leading to platelet clearance via the live^29^,^30^. Whether CD8^+^ T cells also induce platelet desialylation and result in platelet clearance in ITP patients has not been reported.

In the present study, we measured the cytotoxicity of CD8^+^ T cells against autologous platelets, detected platelet desialylation in ITP patients, and cultured CD8^+^ T cells with platelets *in vitro*. The results demonstrated a higher platelet desialylation level in ITP patients with positive cytotoxicity of CD8^+^ T cells than in other ITP patients and controls. CD8^+^ T cells from ITP patients with positive cytotoxicity induced significant platelet desialylation, Neu1 expression on the platelet surface, and platelet phagocytosis by hepatocytes *in vitro*. Moreover, murine experiments showed that pre-activated CD8^+^ splenocytes lowered platelet survival in the circulation and increased platelet phagocytosis in the liver compared to CD8^+^ splenocytes from wild-type (WT) control mice. Taken together, our findings reveal a novel mechanism of platelet destruction in ITP and provide new insights into ITP management.

## Results

### The cytotoxicity of CD8^+^ T cells in ITP patients

**Figure 1:**
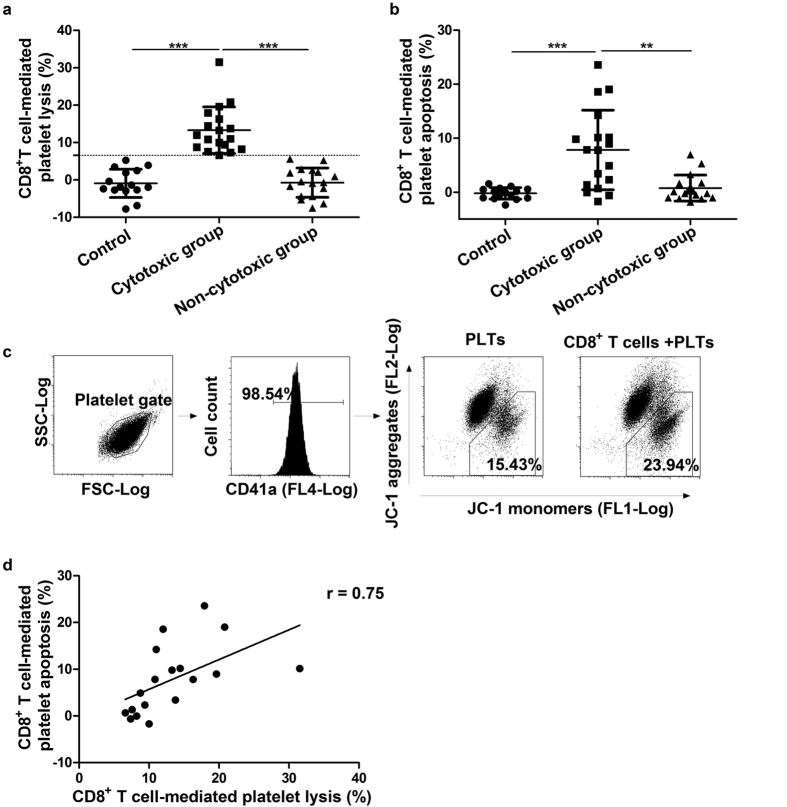
The cytotoxicity of CD8^+^ T cells towards autologous platelets in ITP patients. (**a**) According to platelet lysis mediated by CD8^+^ T cells, ITP patients were assigned to the cytotoxic group (greater than mean plus 2 SD of controls) and the non-cytotoxic group. Dotted line represents the level of mean plus 2 SD of controls. (**b**) Platelet apoptosis mediated by CD8^+^ T cells was significantly higher in the cytotoxic group than in the non-cytotoxic group and controls. (**c**) Representative flow cytometry results from the platelet apoptosis. Platelets were gated according to forward scatter (FSC), side scatter (SSC), and CD41a, then platelet apoptosis was analysed. The dots in the right lower gate represent apoptotic platelets. CD8^+^ T cell-mediated platelet apoptosis was calculated as (apoptosis in the experimental tube - spontaneous apoptosis)/(maximal apoptosis - spontaneous apoptosis) and expressed as a percentage. In the cytotoxic group, CD8^+^ T cells induced significant platelet apoptosis. (**d**) There was a positive correlation between the platelet lysis and platelet apoptosis mediated by CD8^+^ T cells in the cytotoxic group (r = 0.75, *P* < 0.001). Mean ± SD. N = 15 in control group, N = 18 in cytotoxic group and N = 17 in non-cytotoxic group. ^**^P = 0.003 and ^***^P < 0.001.

We measured the cytotoxicity of CD8^+^ T cells first by conducting a CD8^+^ T cell-mediated platelet lysis assay using ^51^Cr labelling. Based on the results from lysis assay, we divided ITP patients into 2 groups. Positive platelet lysis mediated by CD8^+^ T cells (greater than mean plus 2 SD of controls) was assigned to the cytotoxic group (18 of the 35 ITP patients). The other 17 patients were assigned to the non-cytotoxic group (Fig. 1a). Detailed information about the patients in both groups is shown in Table 1. There was no significant difference in platelet counts between the cytotoxic and non-cytotoxic groups.

We also evaluated the cytotoxicity of CD8^+^ T cells by measuring CD8^+^ T cell-mediated platelet apoptosis with JC-1. The results of CD8^+^ T cell-mediated platelet apoptosis assay were in accordance with that of the lysis assay. Platelet apoptosis mediated by CD8^+^ T cells was remarkably higher in the cytotoxic group than in the non-cytotoxic group and controls (cytotoxic vs. non-cytotoxic, 7.81% ± 7.36% vs. 0.77% ± 2.40%, respectively, *P* < 0.001; cytotoxic vs. controls, 7.81% ± 7.36% vs. −0.22% ± 1.05%, respectively, *P* = 0.003; Fig. 1b,c). We further correlated CD8^+^ T cell-mediated platelet lysis with CD8^+^ T cell-mediated apoptosis in the cytotoxic group. The results showed that they were positively correlated (r = 0.75, *P* < 0.001) (Fig. 1d).

### Platelet desialylation elevated in ITP patients with positive cytotoxicity

**Figure 2:**
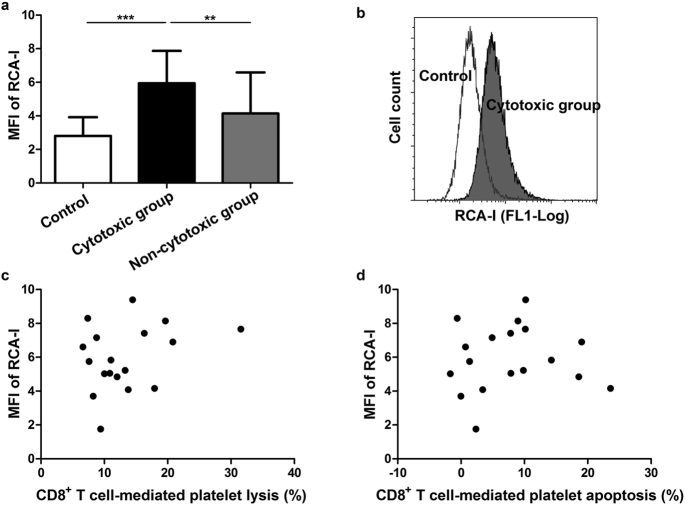
Platelet desialylation in ITP patients with positive cytotoxicity. (**a**) The platelet desialylation level was significantly higher in the cytotoxic group than the non-cytotoxic group and controls. (**b**) Representative flow cytometry results of platelet desialylation in ITP patients with positive cytotoxicity and controls. (**c**) The platelet lysis mediated by CD8^+^ T cells and (**d**) the platelet apoptosis mediated by CD8^+^ T cells was not correlated with the platelet desialylation level in the cytotoxic group. Mean ± SD. N = 15 in control group, N = 18 in cytotoxic group, N = 17 in non-cytotoxic group. ^**^P = 0.008 and ^***^P < 0.001.

We isolated fresh platelets from whole blood samples and detected the platelet desialylation level in ITP patients and controls. The binding level of Ricinus communis agglutinin I (RCA-I) on platelets, which was represented by the mean fluorescence intensity (MFI) of RCA-I, was significantly higher in the cytotoxic group than in the non-cytotoxic group and controls (MFI of RCA-I: cytotoxic vs. non-cytotoxic, 5.95 ± 1.92 vs. 4.14 ± 2.44, respectively, *P* = 0.008; cytotoxic vs. controls, 5.95 ± 1.92 vs. 2.80 ± 1.12, respectively, *P* < 0.001; Fig. 2a,b), revealing a potential association between the cytotoxicity of CD8^+^ T cells and platelet desialylation in ITP. We then analysed the correlation between the cytotoxicity and the platelet desialylation level in the cytotoxic group, and found that CD8^+^ T cell-mediated platelet lysis/apoptosis was not correlated with the MFI of RCA-I of fresh-isolated platelets in ITP patients (Fig. 2c,d).

### CD8^+^ T cells from ITP patients with positive cytotoxicity induced platelet desialylation and Neu1 expression *in vitro*

**Figure 3:**
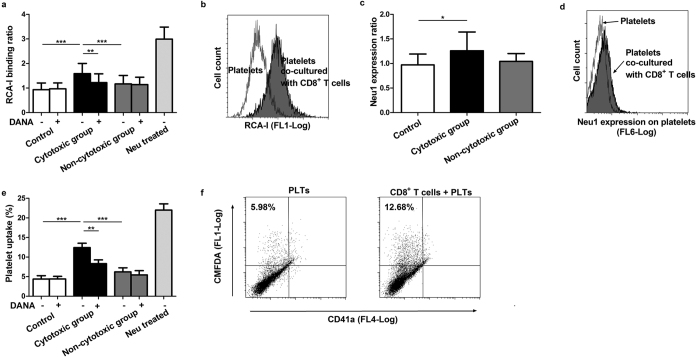
CD8^+^ T cells from ITP patients with positive cytotoxicity induced platelet desialylation and Neu1 expression *in vitro*. (**a**) Desialylation of platelets co-cultured with CD8^+^ T cells from ITP patients in the cytotoxic group (N = 18) was significantly increased compared with platelets co-cultured with CD8^+^ T cells from the non-cytotoxic group (N = 17) and controls (N = 15). Desialylation induced by CD8^+^ T cells from the cytotoxic group was successfully inhibited by neuraminidase inhibitor DANA. Desialylation of normal platelets treated with neuraminidase was also shown (N = 8). (**b**) Representative flow cytometry results of desialylation of platelets cultured with CD8^+^ T cells from ITP patients with positive cytotoxicity and platelets cultured on their own. (**c**) Neu1 expression was significantly increased on platelets co-cultured with CD8^+^ T cells from ITP patients from the cytotoxic group (N = 18) than controls (N = 15). (**d**) Representative flow cytometry results of Neu1 expression on platelets cultured with CD8^+^ T cells from ITP patients with positive cytotoxicity and platelets cultured on their own. (**e**) CD8^+^ T cells from ITP patients in the cytotoxic group (N = 8) induced more significant platelet phagocytosis by HepG2 cells *in vitro* than the rest ITP patients (N = 6) and controls (N = 8), which could be inhibited by DANA. (**f**) The representative results of CMFDA-labeled platelet uptake by HepG2 cells in the cytotoxic group, as detected by flow cytometry as an increase in hepatocyte-associated green fluorescence. Mean ± SD. ^*^P < 0.05, ^**^P < 0.01, and ^***^P < 0.001.

In addition to detecting the desialylation level of fresh platelets, we measured the desialylation of platelets co-cultured with CD8^+^ T cells for 4 hours. RCA-I binding on platelets co-cultured with CD8^+^ T cells from ITP patients in the cytotoxic group was significantly increased compared with those co-cultured with CD8^+^ T cells from the non-cytotoxic group and controls (RCA-I binding ratio: cytotoxic vs. non-cytotoxic, 1.59 ± 0.41 vs. 1.17 ± 0.34, respectively, *P* < 0.001; cytotoxic group vs. controls, 1.59 ± 0.41 vs. 0.94 ± 0.27, respectively, *P* < 0.001). Besides, the RCA-I binding on platelets incubated with neuraminidase was also shown. RCA-I binding induced by CD8^+^ T cells from the cytotoxic group was successfully inhibited by adding neuraminidase inhibitor 2-deoxy-2, 3-didehydro-N-acetylneuraminic acid (DANA) to the cultural medium (RCA-I binding ratio in the cytotoxic group: without DANA vs. with DANA, 1.59 ± 0.41 vs. 1.22 ± 0.36, respectively, *P* = 0.001; Fig. 3a,b). Additionally, DANA did not affect the cytotoxicity of CD8^+^ T cells toward platelets in both ITP patients and controls (data not shown).

Neu1 translocation was previously found to be important for platelet desialylation induced by antiplatelet antibodies in ITP^30^. We speculated that CD8^+^ T cells from ITP patients in the cytotoxic group might induce platelet desialylation through Neu1 translocation. Results showed that Neu1 expression was significantly increased on platelets co-cultured with CD8^+^ T cells from the cytotoxic group than controls (Neu1 expression ratio: cytotoxic group vs. controls: 1.19 ± 0.23 vs. 0.95 ± 0.21, respectively, *P* = 0.029). However, in the non-cytotoxic group, Neu1 expression did not change significantly (Fig. 3c,d).

### CD8^+^ T cells from ITP patients with positive cytotoxicity induced platelet phagocytosis by hepatocytes *in vitro*

*In vitro* HepG2 based platelet ingestion assay was conducted to study the platelet phagocytosis by hepatocytes. We further enrolled 14 ITP patients (8 in cytotoxic group and 6 in non-cytotoxic group) and 8 controls. Results showed that platelet ingestion by HepG2 cells was significantly higher after platelets were co-cultured with CD8^+^ T cells from ITP patients in the cytotoxic group than CD8^+^ T cells from the non-cytotoxic group and controls (cytotoxic group vs. non-cytotoxic group: 12.44% ± 3.09% vs. 6.21% ± 2.55%, respectively, *P* < 0.001; cytotoxic group vs. controls: 12.44% ± 3.09% vs. 4.38% ± 2.44%, respectively, *P* < 0.001), which could be inhibited by neuraminidase inhibitor DANA (without DANA vs. with DANA, 12.44% ± 3.09% vs. 8.32% ± 2.70%, respectively, *P* = 0.003, Fig. 3e,f). The results indicated that CD8^+^ T cells from ITP patients in the cytotoxic group induced platelet phagocytosis by hepatocytes via desialylation.

### CD8^+^ T cells from immunized CD61 knockout (KO) mice induced platelet desialylation and Neu1 expression *in vitro*

**Figure 4:**
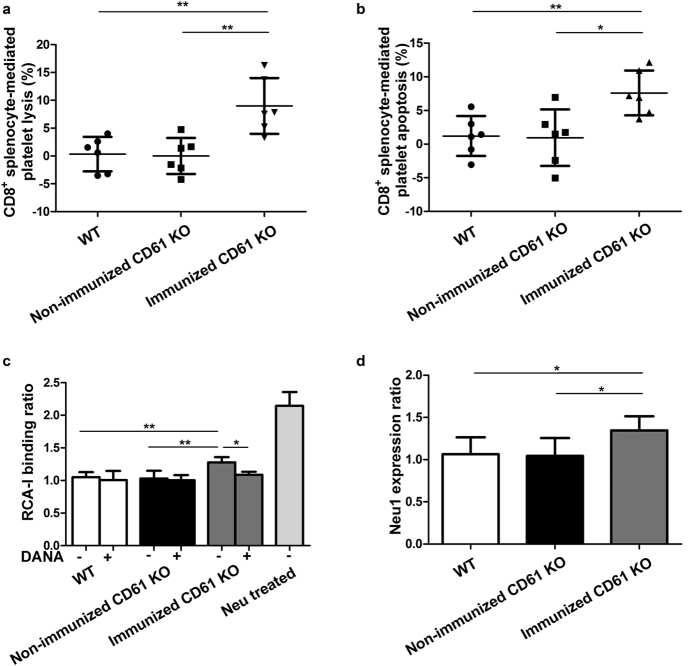
Murine CD8^+^ splenocytes induced platelets desialylation and Neu1 expression on platelets. (**a,b**) Platelet lysis (**a**) and apoptosis (**b**) induced by CD8^+^ splenocytes from immunized CD61 KO mice were significantly higher than WT and non-immunized CD61 KO mice. (**c**) RCA-1 binding on platelets co-cultured with CD8^+^ splenocytes from immunized CD61 KO mice was significantly increased compared with co-cultured with CD8^+^ splenocytes from WT and non-immunized CD61 KO mice. This RCA-1 binding was inhibited by DANA. (**d**) Neu1 expression on platelets co-cultured with CD8^+^ splenocytes from immunized CD61 KO mice was significantly increased compared to platelets co-cultured with CD8^+^ splenocytes from WT and non-immunized CD61 KO mice. N = 5–6 in each group. ^*^P < 0.05 and ^**^P < 0.01.

The cytotoxicity of CD8^+^ splenocytes towards platelets from immunized CD61 KO mice was detected by measuring the CD8^+^ splenocyte-mediated platelet lysis and apoptosis using ^51^Cr release assay and JC-1 staining respectively. CD8^+^ splenocytes from WT and non-immunized CD61 KO mice were used as control groups. The results showed that both the platelet lysis and apoptosis mediated by CD8^+^ splenocytes from immunized CD61 KO mice were significantly higher than control groups (CD8^+^ splenocyte-mediated platelet lysis: immunized CD61 KO mice vs. WT mice, 8.99% ± 5.00% vs. 0.34% ± 3.09%, respectively, *P* = 0.005, immunized CD61 KO mice vs. non-immunized CD61 KO mice, 8.99% ± 5.00% vs. 0.00% ± 3.23%, respectively, *P* = 0.004; CD8^+^ splenocyte-mediated platelet apoptosis: immunized CD61 KO mice vs. WT mice, 7.60% ± 3.33% vs. 1.20% ± 2.97%, respectively, *P* = 0.006, immunized CD61 KO mice vs. non-immunized CD61 KO mice, 7.60% ± 3.33% vs. 0.96% ± 4.20%, respectively, *P* = 0.010) (Fig. 4a,b). Then the platelet desialylation level and Neu1 expression on platelets were analysed. Murine *in vitro* results were consistent with the results obtained with human platelets. RCA-I binding on platelets co-cultured with CD8^+^ splenocytes from immunized CD61 KO mice was significantly increased compared to platelets co-cultured with CD8^+^ splenocytes from WT and non-immunized CD61 KO mice (RCA-I binding ratio: immunized CD61 KO mice vs. WT mice, 1.28 ± 0.08 vs. 1.05 ± 0.08, respectively, *P* = 0.002; immunized CD61 KO mice vs. non-immunized CD61 KO mice, 1.28 ± 0.08 vs. 1.03 ± 0.12, respectively, *P* = 0.005). RCA-1 binding induced by these CD8^+^ splenocytes was inhibited by DANA (without DANA vs. with DANA: 1.28 ± 0.08 vs. 1.09 ± 0.05, *P* = 0.012; Fig. 4c). Besides, the RCA-I binding on platelets incubated with neuraminidase was also shown. Furthermore, after co-cultured with CD8^+^ splenocytes from immunized CD61 KO mice, Neu1 expression on platelets was significantly increased than that on platelet co-cultured with CD8^+^ splenocytes from WT and non-immunized CD61 KO mice (Neu1 expression ratio: immunized CD61 KO mice vs. WT mice, 1.35 ± 0.17 vs. 1.06 ± 0.20, respectively*, P* = 0.042, immunized CD61 KO mice vs. non-immunized CD61 KO mice, 1.35 ± 0.17 vs. 1.04 ± 0.21, respectively, *P* = 0.037; Fig. 4d).

### CD8^+^ T cells decreased platelet survival rate in circulation *in vivo*

**Figure 5:**
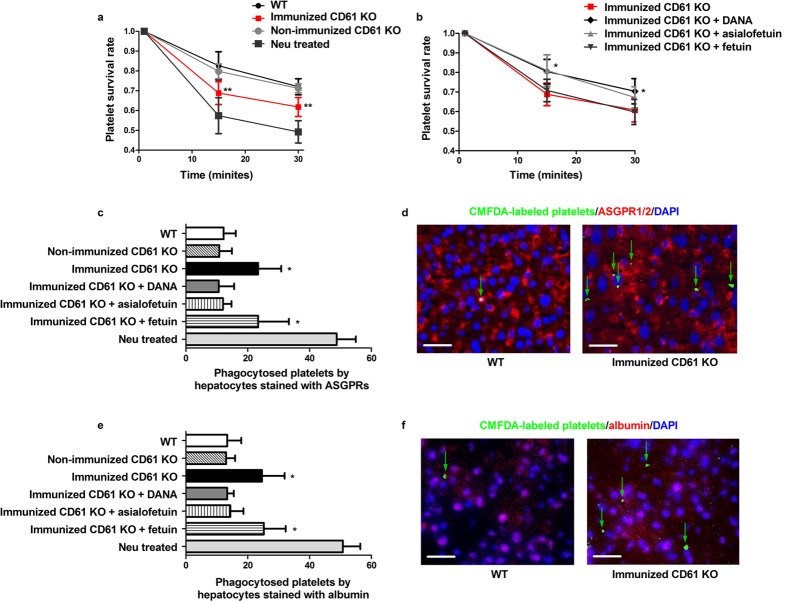
CD8^+^ splenocytes caused decreased platelet survival and increased platelet phagocytosis in the liver. (**a**) Platelet survival in the circulation measured by the percentage of CMFDA-labeled platelets in whole blood samples that were harvested 1 (baseline), 15, and 30 min after platelet transfusion. Platelet survival rate was much lower in the group in which platelets were co-cultured with CD8^+^ splenocytes from immunized CD61 KO mice than those co-cultured with CD8^+^ splenocytes from WT and non-immunized mice. The survival rate of neuraminidase-treated platelets was also shown. (**b**) The survival rate of platelets co-cultured with CD8^+^ splenocytes from immunized CD61 KO mice could be significantly improved by DANA and ASGPRs blocker asialofetuin. (**c**) CMFDA-labeled platelets incubated with CD8^+^ splenocytes from immunized CD61 KO mice experienced more phagocytosis by ASGPRs-expressing hepatocytes in the liver than control platelets. This phagocytosis was significantly alleviated by DANA and asialofetuin. (**d**) Representative images of *in vivo* platelet (green) phagocytosis by ASGPRs-expressing hepatocytes (red) detected by immunofluorescence. Nuclei were counterstained with DAPI (blue). Images were taken using an IX71-Olympus inverted fluorescence microscope with LUCPlanFLN 20×/0.45 Ph1 objective lens and acquired by cellSens Standard software. ImageJ software was used to merge channels and analyse images. Scale bars represent 20 μm. (**e,f**) Albumin was also used to stain hepatocytes (red) and similar results were obtained. Mean ± SD. N = 5 in each group, ^*^P < 0.05 and ^**^P < 0.01.

Murine *in vivo* experiments were conducted to study the effect of CD8^+^ splenocytes on platelet survival in the circulation. We found that the survival of 5-chloromethylfluorescein diacetate (CMFDA)-labeled platelets in whole blood was significantly lower after co-culture with CD8^+^ splenocytes from immunized CD61 KO mice compared to co-culture with CD8^+^ splenocytes from WT and non-immunized CD61 KO mice (at 15 min, immunized CD61 KO mice vs. WT mice, 0.69 ± 0.06 vs. 0.83 ± 0.07, respectively, *P* = 0.004, immunized CD61 KO mice vs. non-immunized CD61 KO mice, 0.69 ± 0.06 vs. 0.80 ± 0.04, respectively, *P* = 0.007; at 30 min, immunized CD61 KO mice vs. WT mice, 0.61 ± 0.06 vs. 0.72 ± 0.04, respectively, *P* = 0.006, immunized CD61 KO mice vs. non-immunized CD61 KO mice, 0.61 ± 0.06 vs. 0.71 ± 0.02, respectively, *P* = 0.004; Fig. 5a). These findings demonstrated that antigen-primed CD8^+^ T cells reduced the survival of platelets in the circulation, which might be a novel mechanism of thrombocytopenia in ITP patients.

To better elucidate whether platelet desialylation contributes to the decreased platelet survival caused by CD8^+^ splenocytes, we added neuraminidase inhibitor DANA to the co-culture medium. DANA significantly improved the survival of platelets co-cultured with CD8^+^ splenocytes from immunized CD61 KO mice (without DANA vs. with DANA: at 15 min, 0.69 ± 0.06 vs. 0.80 ± 0.06, *P* = 0.012; at 30 min, 0.61 ± 0.06 vs. 0.70 ± 0.06, *P* = 0.016, respectively; Fig. 5b).

Because previous reports indicated that desialylated platelets were cleared by ASGPR-expressing hepatocytes^12^, we also co-injected asialofetuin (ASGPRs competitor) or fetuin (the non-specific control to asialofetuin) into mice with transfused platelets. The co-injection of asialofetuin significantly improved the survival of platelets co-cultured with CD8^+^ splenocytes from immunized CD61 KO mice (without asialofetuin vs. asialofetuin: at 15 min, 0.69 ± 0.06 vs. 0.81 ± 0.08, *P* = 0.009; at 30 min, 0.61 ± 0.06 vs. 0.67 ± 0.05, *P* = 0.095, respectively). Fetuin did not show a significant effect (Fig. 5b).

### CD8^+^ T cells increased platelet clearance via the liver *in vivo*

Platelet clearance via the liver was measured in mice by immunofluorescence. CMFDA-labeled platelets co-cultured with CD8^+^ splenocytes from immunized CD61 KO mice experienced more phagocytosis by ASGPRs-expressing hepatocytes in the liver when transfused into WT mice compared to platelets co-cultured with CD8^+^ splenocytes from WT and non-immunized CD61 KO mice (23.40 ± 7.40 vs. 12.10 ± 3.96, *P* = 0.011). This phagocytosis was significantly alleviated by DANA (without DANA vs. with DANA, 23.40 ± 7.40 vs. 10.60 ± 5.03, respectively, *P* = 0.005) and asialofetuin (without asialofetuin vs. with asialofetuin, 23.40 ± 7.40 vs. 12.00 ± 2.74, *P* = 0.010; Fig. 5c,d). Moreover, we used albumin to stain hepatocytes and similar results were obtained (Fig. 5e,f). These data suggest that CD8^+^ T cells result in platelets desialylation that leads to platelet phagocytosis in the liver in ITP.

## Discussion

In the present study, we found a novel mechanism of platelet clearance caused by CD8^+^ T cells in ITP. Through both *in vitro* and *in vivo* experiments, we demonstrated that CD8^+^ T cells induced platelet desialylation and Neu1 expression on human and murine platelets, and subsequently resulted in platelet clearance in the liver in a mouse model of ITP.

Cell-mediated cytotoxic lysis of platelets has been shown to contribute to the increased platelet destruction in ITP^1^,^5^,^31^,^32^. In the present study, positive CD8^+^ T cell-mediated platelet lysis *in vitro* was found in more than a half of ITP patients. CD8^+^ T cell-mediated platelet apoptosis was also elevated in these patients. They were positively correlated. Several studies and our research found that CD8^+^ T cells could exert their cytotoxicity towards platelets by causing direct platelet lysis or apoptosis when they were co-cultured with platelets *in vitro*^1^,^2^,^33^,^34^. Activated CD8^+^ T cells kill platelets mainly through perforin/granzymes-mediated pathway^1^,^35^,^36^. In this study, the mean CD8^+^ T cell-mediated platelet lysis was 11.54% and the mean CD8^+^ T cell-mediated platelet apoptosis was 7.81% in the cytotoxic group. The results were relatively low values compared with tumor targets^37^,^38^, but were in line with previous studies about ITP^1^,^2^,^33^,^34^. It might due to the low effector/target ratio we used in this manuscript and the different immunologic conditions between patients with tumors and those with autoimmune diseases. The number of antigen-specific CD8^+^ CTLs is relatively small and insufficient to lyse a mass of platelets in ITP. Thus, there must be another mechanism(s) contributing to CD8^+^ T cell-mediated platelet clearance.

Platelet desialylation is an important avenue for platelet clearance. It is responsible for thrombocytopenia in many diseases and pathological situations^17^,^19^,^21^,^27^,^39^. However, its role in autoimmune diseases remains not well elucidated. A previous case report described that a pediatric chronic ITP patient was administered neuraminidase inhibitor oseltamivir phosphate to treat an influenza A virus infection and, surprisingly, her platelet counts progressively increased without other blood products or agents^40^. Our recent report also showed that oseltamivir phosphate was successfully used to treat an adult primary ITP patient who responded poorly to conventional therapies, including corticosteroids, intravenous immunoglobulin, recombinant human thrombopoietin, rituximab, danazol and vindesine^41^. The alleviation of thrombocytopenia in these two cases suggested that neuraminidase inhibition might be an effective treatment for some ITP patients and raised the question of whether platelet desialylation contributes to thrombocytopenia in ITP. Later, Li *et al*. demonstrated that anti-GPIbα autoantibodies induced platelet desialylation and led to platelet clearance in the liver via hepatocyte ASGPRs in a mouse model of ITP^29^,^30^. This novel clearance mechanism was completely different from the classical macrophage phagocytosis in the spleen. Because antiplatelet autoimmunity in ITP is mainly caused by both antiplatelet autoantibodies and CD8^+^ T cells, it is possible that CD8^+^ T cells also induce platelet desialylation.

In the present study, we found that desialylation of fresh platelets from ITP patients with positive cytotoxicity of CD8^+^ T cells was significantly higher than non-cytotoxic ITP patients and controls, suggesting a possible role for desialylation in CD8^+^ T cell-mediated platelet clearance in ITP. However, there was no correlation between the cytotoxicity of CD8^+^ T cells and the desialylation level of fresh-isolated platelets in the cytotoxic group. Anti-GPIb but not anti-GPIIb/IIIa has been shown to cause platelet desialylation in ITP^30^. Antiplatelet antibodies and CD8^+^ T cells co-act in platelet destruction in many ITP patients. A previous study showed that CD8^+^ CTL-mediated platelet destruction was predominant in antibodies negative ITP patients^33^. The presence of antiplatelet antiantibodies might interfere the analysis of the association between CD8^+^ T cells and platelet desialylation. To further elucidate the role of the cytotoxicity of CD8^+^ T cells on platelet desialylation, we conducted *in vitro* co-culture of CD8^+^ T cells and platelets, and demonstrated that CD8^+^ T cells from ITP patients with positive cytotoxicity induced platelet desialylation. Our results indicated that CD8^+^ T cells caused platelet desialylation in ITP and helped to explain CD8^+^ CTLs contribute to destruction of the huge number of platelets in ITP despite their limited population. These results further confirm the pathogenic diversity of ITP^32^.

Neuraminidases are sialic acid-releasing exoglycosidases that catalyze platelet desialylation. Treating platelets by administering neuraminidases *in vitro* or by direct injecting neuraminidases into animals result in evident platelet desialylation^9^,^42^,^43^. Vertebrate neuraminidases are a family of four enzymes that possess differential distribution and particular substrate preferences (Neu1-4)^44^,^45^. Among them, Neu1 is a lysosomal neuraminidase with narrow substrate specificity that hydrolyzes sialic acids from glycoproteins preferentially^25^. Studies have revealed that Neu1 is stored in a specialized compartment inside platelets and is undetectable on the surface of resting platelets, but has been found to be translocated to the platelet surface or released in some situations^17^,^29^,^30^. Anti-GPIb antibodies have been shown to cause Neu1 translocation^30^. Our results indicated that CD8^+^ T cells from ITP patients with positive cytotoxicity induced Neu1 expression on the platelet surface compared to CD8^+^ T cells from non-cytotoxic ITP patients and controls. Furthermore, we added neuraminidase inhibitor DANA to the cultural medium to inhibit the activity of Neu1 and found that DANA successfully inhibited the increased platelet desialylation caused by CD8^+^ T cells but did not affect the cytotoxicity fo CD8^+^ T cells in ITP. Taken together, these results suggested that CD8^+^ T cells caused Neu1 expression on platelet surface.

Desialylated platelets were previously shown to be phagocytosed by ASGPRs-expressing hepatocytes in the liver and cleared from the circulation^17^,^27^. Therefore, we hypothesized that CD8^+^ T cells might cause platelet clearance in the liver in ITP. *In vitro* HepG2 based platelet ingestion assay and murine experiments were conducted to test our hypothesis. *In vitro* HepG2 based platelet ingestion assay showed that CD8^+^ T cells from ITP patients with positive cytotoxicity accelerated platelet phagocytosis by hepatocytes, which could be inhibited by DANA. Additionally, murine experiments obtained similar results. The immunization of CD61 KO mice was performed according to the method described by Chow L *et al*.^46^. After immunization, CD8^+^ splenocytes were antigen-primed against WT platelets and exerted their cytotoxicity. For platelet survival in circulation, we found that the platelets co-cultured with CD8^+^ splenocytes from immunized CD61 KO mice experienced a decreased survival rate compared to controls after transfused into WT mice. For platelet phagocytosis in the liver, we found that platelets underwent more phagocytosis by ASGPR1/2-labelled hepatocytes after co-culture with CD8^+^ splenocytes from immunized CD61 KO mice. ASGPRs (made up of ASGPR1 and ASGPR2 subunits) are a type of lectin and mediate the recognition, binding, and clearance of asialoglycoproteins^12^,^28^. They are predominantly expressed in the liver and are often used as markers of hepatocytes^47^. Moreover, we also used albumin to stain hepatocytes to study the platelet phagocytosis in the liver and achieved similar results. Previous studies showed that both DANA and asialofetuin significantly improved the survival and decreased the clearance of desialylated platelets of cold-stored platelets^27^ and a passive murine model on an anti-GPIb-mediated ITP^30^. We found that addition of DANA to the culture medium or co-injection of ASGPRs competitor asialofetuin significantly improved platelet survival in the circulation and attenuated platelet clearance via the liver caused by CD8^+^ splenocytes. These findings indicated that CD8^+^ T cells caused platelet clearance via platelet desialylation in the liver in ITP. Inhibiting platelet desialylation decreased platelet clearance in the liver caused by CD8^+^ splenocytes but, did not affect CD8^+^ T cell-mediated platelet lysis. Inhibiting platelet desialylation and/or blocking hepatocyte ASGPRs may alleviate thrombocytopenia in ITP patients, especially those with positive cytotoxicity of CD8^+^ T cells and those who respond poorly to conventional first- and second-line therapies.

In conclusion, our results suggest that CD8^+^ T cells result in platelet clearance via the liver by inducing platelet desialylation. Inhibiting platelet desialylation or blocking the clearance of desialylated platelets via the liver may be novel therapeutic strategies for ITP patients.

## Methods

### Patients and controls

Thirty-five newly-diagnosed ITP patients (21 female and 14 male; age range 18 to 74 years, median 42 years) were enrolled in the Department of Hematology, Qilu Hospital, Shandong University, Jinan, China. Platelet counts ranged from 8 to 41 × 10^9^/L, with a median count of 16 × 10^9^/L. All patients met the diagnostic criteria of ITP^48^. None of the patients had received ITP-specific treatment before sampling. Fifteen healthy controls (9 females and 6 males; age range 19 to 72 years, median age 40 years) were enrolled. The platelet counts ranged from 165 to 287 × 10^9^/L, with a median count of 236 × 10^9^/L. An additional 14 newly-diagnosed ITP patients (9 females and 5 males; age range 21 to 69 years, median 40 years, platelet range, 12 to 38 × 10^9^/L, median 19 × 10^9^/L) and 8 controls (5 females and 3 males; age range 19 to 65 years, median 38 years, platelet range, 176 to 295 × 10^9^/L, median 210 × 10^9^/L) were enrolled for *in vitro* platelet phagocytosis assay. Enrollment took place between February 2014 and April 2016. Informed consent was gained from each participant. Ethical approval was given by the Medical Ethical Committee of Qilu Hospital, Shandong University. All experiments were carried out in accordance with the approved guidelines.

### Preparation of human platelets and CD8^+^ T cells

Ethylene diamine tetraacetic acid anticoagulated peripheral blood was obtained from ITP patients and controls by venipuncture. Platelet-rich plasma was isolated by centrifugation at 200 *g* for 10 minutes. Fresh platelets and platelet-poor plasma were separated from platelet-rich plasma by centrifugation at 850 *g* for 5 minutes. Peripheral blood mononuclear cells were prepared from whole blood by Ficoll-Hypaque density gradient centrifugation. CD8^+^ T cells from peripheral blood mononuclear cells were obtained by positive selection with CD8 magnetic microbeads (Miltenyi Biotec, Bergisch Gladbach, Germany). The purity of CD8^+^ cells was always greater than 95%, as analysed by flow cytometry. All cells were washed free of plasma.

### Neuraminidase treatment

To show the maximal platelet desialylation level, human and murine platelets (10^8^/mL) were treated with 2.5 mU of α2-3,6,8,9-sialidase from *Arthrobacter ureafaciens* (Calbiochem) at 37 °C for 15 min.

### *In vitro* co-culture of human CD8^+^ T cells and platelets

CD8^+^ T cells and platelets were co-cultured as previously described^34^. Briefly, CD8^+^ T cells and autologous platelets were used as effector and target cells, respectively. They were diluted in RPMI 1640 (Invitrogen, Grand Island, NY, USA) culture medium containing 10% heat-inactivated fetal calf serum (Hyclone, Logan, Utah, USA) and 320 ng/mL anti-CD3 antibody (eBioscience, San Diego, CA, USA) to achieve a final effector/target ratio of 1:10, and co-cultured at 37°C for 4 hours. Neuraminidase inhibitor DANA (Calbiochem, Billerica, MA, USA) was added to a final concentration of 1 mM.

### Assay of CD8^+^ T cells-mediated platelet lysis

Human platelets were radiolabeled with ^51^Cr (PerkinElmer, Waltham, USA) as previously described^49^ and co-cultured with CD8^+^ T cells for 4 hours. Thereafter, the supernatants were obtained by centrifugation and counted in a gamma counter. According to the method used by Olsson *et al*.^1^, CD8^+^ T cell-mediated platelet lysis was calculated as (lysis in experimental tube - spontaneous lysis)/(maximal lysis - spontaneous lysis) and expressed as a percentage. Spontaneous lysis was measured in the tubes that contained only culture media and platelets. Maximal lysis was determined in the tubes that contained 1% Triton X-100 (Sigma-Aldrich, St. Louis, MO, USA) and platelets.

### Antiplatelet autoantibody assay

Plasma samples were stored at −80°C until use. The modified antibody-specific immobilization of platelet antigens (MAIPA) was used to detect the specific anti-GPIb and/or anti-GPIIb/IIIa autoantibodies as described by Hou *et al*.^50^.

### Flow cytometry

To detect platelet desialylation, PE-Cy5-labelled anti-human CD41 monoclonal antibodies (20 μL per test) (BD Bioscience, San Jose, CA. USA) were used to label human platelets. FITC-labelled RCA-I (5 μg/mL) (Vector Laboratories, Burlingame, CA, USA) was used to analyse β-galactose exposure on platelet surfaces. Platelets (1 × 10^6^) were incubated with anti-CD41 and RCA-I for 20 minutes, washed twice and resuspended. Neu1 expression on platelet surface was measured with rabbit anti-Neu1 IgG antibody (Santa Cruz, Dallas, Texas, USA; 1:50 dilution) and detected with Alexa Fluor 647 secondary antibody (Abcam, Hong Kong, China; 1:2000 dilution) as described by June *et al*.^30^. Platelet apoptosis was determined by a mitochondrial membrane potential assay kit with JC-1 (Beyotime, Nantong, China) according to the manufacturer’s recommendations^51^,^52^. Cell acquisition was performed on a Beckman Gallios™ Flow Cytometer (Beckman Coulter, Fullerton, CA, USA). A total of 10,000 events were analysed using Gallios™ Cytometry List Mode Data Acquisition & Analysis Software (Beckman Coulter).

The MFI of RCA-I and Neu1 represented the levels of platelet desialylation and Neu1 expression respectively. In co-culture experiments, the RCA-I binding ratio and the Neu1 expression ratio were applied. The RCA-I binding ratio was calculated as (MFI of RCA-I in the experimental tube)/(MFI of RCA-I in the tube containing only culture media and platelets). The Neu1 expression ratio was calculated as (MFI of Neu1 in the experimental tube)/(MFI of Neu1 in the tube containing only culture media and platelets). CD8^+^ T cell-mediated platelet apoptosis was calculated as (apoptosis in the experimental tube - spontaneous apoptosis)/(maximal apoptosis - spontaneous apoptosis) and expressed as a percentage. Maximal apoptosis was measured in the tubes that contained 10 μM carbonyl cyanide 3-chlorophenylhydrazone (CCCP, Beyotime) and platelets.

### *In vitro* HepG2 based platelet ingestion assay

The HepG2 cells were cultured in DMEM (Invitrogen) culture medium containing 10% heat-inactivated fetal calf serum, transferred to 24-well plates (10^6^ per well) and allowed to adhere for 24 h. Platelets were stained with 5 μM of CMFDA (Invitrogen, Grand Island, NY), co-cultured with CD8^+^ T cells with or without DANA as described above, separated through centrifugation and added to plates (10^7^ per well). Hepatocytes and platelets were incubated for 30 min at 37 °C with gentle agitation. Then the HepG2 monolayers were washed 3 times by PBS, dissociated from the wells with trypsin (GIBCO Invitrogen) and stained with anti-CD41a. Platelet ingestion was quantified by flow cytometry. Single CMFDA^+^ events stand for hepatocytes with ingested platelets. CD41a^+^CMFDA^+^ events represent hepatocytes with adherent platelets^12^,^23^,^27^.

### Mice

WT C57BL/6 mice (6–8 weeks) were purchased from the Centre for New Drug Evaluation of Shandong University. CD61 KO C57BL/6 mice were kindly provided by Professor Junling Liu (Department of Biochemistry and Molecular Cell Biology, Shanghai Key Laboratory of Tumor Microenvironment and Inflammation, Shanghai Jiao Tong University School of Medicine, Shanghai, China) and bred in the Animal Centre of Qilu Hospital, Shandong University. The immunization of CD61 KO mice with WT platelets were conducted as previously described by Chow *et al*.^46^. All experimental protocols were approved by the Medical Ethical Committee of Qilu Hospital, Shandong University and undertaken in accordance with the Institutional Guidelines for the Care and Use of Laboratory Animals.

### *In vitro* co-culture of murine CD8^+^ splenocytes and platelets

For the preparation of murine platelets and CD8^+^ splenocytes, mice were anesthetized and blood was collected into phosphate-buffered saline (PBS) containing citrate/phosphate, dextrose, adenine buffer by retro-orbital eye bleed. Platelets were prepared through centrifugation. Spleens were removed from mice and crushed through a mesh filter in PBS to make a splenocyte suspension. The suspension was washed by centrifugation at 400 *g* for 15 minutes for 3 times to obtain splenocytes. CD8^+^ splenocytes were separated using murine CD8 magnetic microbeads (Miltenyi Biotec). Murine CD8^+^ splenocytes and platelets were co-cultured. CD8^+^ T cell-mediated platelet lysis and apoptosis were detected by ^51^Cr release assay and JC-1 staining respectively, and platelet desialylation and Neu1 expression levels of platelets were detected by flow cytometry similarly to human.

### *In vivo* platelet survival

A total of 10^8^ WT murine platelets were labelled with 5 μM of CMFDA and co-cultured with CD8^+^ splenocytes, separated through centrifugation, washed and injected into WT mice through the caudal vein. To test the role of hepatocyte ASGPRs in platelet clearance caused by CD8^+^ splenocytes, a bolus of 10 mg ASGPRs competitor asialofetuin or a non-specific control fetuin (Sigma-Aldrich) was injected via the caudal vein into WT mice 2 min before the platelet transfusion. Following transfusion, 5 mg asialofetuin or fetuin was injected at 10 and 20 min. To determine platelet survival in the circulation, whole blood samples were obtained at time points of 1 min (baseline), 15 min and 30 min. The percentage of CMFDA positive platelets in whole blood was determined by flow cytometry. The 1-min values of CMFDA positive platelets were set at 100%.

### Immunofluorescence

Thirty minutes post platelet injection, mice were anesthetized and drained of blood through retro-orbital eye bleeding. Murine liver was harvested and snap-frozen in liquid nitrogen. Frozen tissues were sectioned (5 μm) and fixed onto slides in ice-cold acetone. The slides were subsequently blocked in 10% goat serum (Zhongshan Golden Bridge Bio-technology, Beijing, China) and incubated with primary rabbit anti-ASPGR1/2 (Santa Cruz, 1:50 dilution) or rabbit anti-albumin (Santa Cruz, 1:20 dilution) overnight at 4 °C. Then, sections were stained with goat anti-rabbit tetraethyl rhodamine isothiocyanate (TRITC) secondary antibody (Zhongshan Golden Bridge Bio-technology, 1:50 dilution) for 2 hours. Cell nuclei were stained with 5 μg/mL DAPI (Beyotime). Sections were imaged using Olympus inverted fluorescence microscope. Images were analysed using Adobe Photoshop 5 and Image J. Platelets that were phagocytosed by hepatocytes were counted in 5 randomized fields (200×).

### Statistics

Data are expressed as mean ± SD. Statistical significance was determined by Student’s *t* test and analysis of variance (ANOVA), unless the data were not normally distributed, in which case the Mann-Whitney U test and the Kruskal-Wallis test were used. Pearson correlation was used for correlation analysis unless the data were not normally distributed, in which case Spearman correlation analysis was conducted. *P* < 0.05 was considered statistically significant.

## Additional Information

**How to cite this article**: Qiu, J. *et al*. CD8^+^ T cells induce platelet clearance in the liver via platelet desialylation in immune thrombocytopenia. *Sci. Rep.* **6**, 27445; doi: 10.1038/srep27445 (2016).

## Figures and Tables

**Table 1:** Data for the 35 ITP patients.

PatientNo.	Sex	Age(years)	Course ofITP (days)	Antiplateletautoantibodies		Platelets(10^9^/mL)
				Anti-GPIb	Anti-GPIIb/IIIa	
Cytotoxic group						
1	M	36	5	−	+	21
2	F	24	3	+	−	9
3	F	19	27	−	−	12
4	M	56	20	−	−	17
5	F	47	55	+	+	15
6	M	27	7	−	−	26
7	F	54	30	−	−	28
8	F	65	7	−	−	8
9	F	60	13	+	−	9
10	F	43	24	−	−	12
11	M	51	16	−	−	14
12	M	41	28	−	+	10
13	F	51	50	−	−	36
14	M	72	20	+	+	14
15	F	21	4	−	−	36
16	F	26	28	+	+	16
17	M	20	2	+	−	11
18	F	67	15	−	+	41
Non-cytotoxic group						
19	F	42	3	−	−	8
20	M	25	14	−	−	29
21	M	48	26	+	−	11
22	F	29	15	−	−	16
23	F	40	55	−	−	38
24	M	20	5	−	+	23
25	F	60	7	+	+	22
26	M	66	18	+	+	16
27	M	18	15	−	+	10
28	F	29	3	+	+	14
29	F	74	20	+	−	10
30	F	41	35	−	−	15
31	M	25	6	−	+	28
32	F	56	14	+	−	9
33	F	50	35	−	−	34
34	F	52	20	−	+	19
35	M	41	55	+	−	20

F: female; M: male.

## References

[b1] Olsson B. . T-cell-mediated cytotoxicity toward platelets in chronic idiopathic thrombocytopenic purpura. Nature medicine 9, 1123–1124, 10.1038/nm921 (2003). 12937414

[b2] Zhang F. . Cell-mediated lysis of autologous platelets in chronic idiopathic thrombocytopenic purpura. European journal of haematology 76, 427–431, 10.1111/j.1600-0609.2005.00622.x (2006). 16480433

[b3] Nugent D., McMillan R., Nichol J. L. & Slichter S. J. Pathogenesis of chronic immune thrombocytopenia: increased platelet destruction and/or decreased platelet production. British journal of haematology 146, 585–596, 10.1111/j.1365-2141.2009.07717.x (2009). 19466980

[b4] Cines D. B. & Blanchette V. S. Immune thrombocytopenic purpura. The New England journal of medicine 346, 995–1008, 10.1056/NEJMra010501 (2002). 11919310

[b5] Cooper N. & Bussel J. The pathogenesis of immune thrombocytopaenic purpura. British journal of haematology 133, 364–374, 10.1111/j.1365-2141.2006.06024.x (2006). 16643442

[b6] Li S. . CD8+ T cells suppress autologous megakaryocyte apoptosis in idiopathic thrombocytopenic purpura. British journal of haematology 139, 605–611, 10.1111/j.1365-2141.2007.06737.x (2007). 17979946

[b7] Morell A. G., Gregoriadis G., Scheinberg I. H., Hickman J. & Ashwell G. The role of sialic acid in determining the survival of glycoproteins in the circulation. The Journal of biological chemistry 246, 1461–1467 (1971). 5545089

[b8] Hoffmeister K. M. The role of lectins and glycans in platelet clearance. Journal of thrombosis and haemostasis: JTH 9 Suppl 1, 35–43, 10.1111/j.1538-7836.2011.04276.x (2011). 21781240 PMC4303235

[b9] Greenberg J., Packham M. A., Cazenave J. P., Reimers H. J. & Mustard J. F. Effects on platelet function of removal of platelet sialic acid by neuraminidase. Laboratory investigation; a journal of technical methods and pathology 32, 476–484 (1975). 1127870

[b10] Hoffmeister K. M. . The clearance mechanism of chilled blood platelets. Cell 112, 87–97 (2003). 12526796 10.1016/s0092-8674(02)01253-9

[b11] Josefsson E. C., Gebhard H. H., Stossel T. P., Hartwig J. H. & Hoffmeister K. M. The macrophage alphaMbeta2 integrin alphaM lectin domain mediates the phagocytosis of chilled platelets. The Journal of biological chemistry 280, 18025–18032, 10.1074/jbc.M501178200 (2005). 15741160

[b12] Rumjantseva V. . Dual roles for hepatic lectin receptors in the clearance of chilled platelets. Nature medicine 15, 1273–1280, 10.1038/nm.2030 (2009). PMC4428152 19783995

[b13] Paris L. L., Chihara R. K., Sidner R. A., Tector A. J. & Burlak C. Differences in human and porcine platelet oligosaccharides may influence phagocytosis by liver sinusoidal cells *in vitro*. Xenotransplantation 19, 31–39, 10.1111/j.1399-3089.2011.00685.x (2012). 22360751

[b14] Jhang J. S. & Spitalnik S. L. Glycosylation and cold platelet storage. Current hematology reports 4, 483–487 (2005). 16232387

[b15] Grewal P. K. . The Ashwell receptor mitigates the lethal coagulopathy of sepsis. Nature medicine 14, 648–655, 10.1038/nm1760 (2008). PMC2853759 18488037

[b16] Rumjantseva V. & Hoffmeister K. M. Novel and unexpected clearance mechanisms for cold platelets. Transfusion and apheresis science: official journal of the World Apheresis Association: official journal of the European Society for Haemapheresis 42, 63–70, 10.1016/j.transci.2009.10.008 (2010). PMC4303245 19932055

[b17] Jansen A. J. . Desialylation accelerates platelet clearance after refrigeration and initiates GPIbalpha metalloproteinase-mediated cleavage in mice. Blood 119, 1263–1273, 10.1182/blood-2011-05-355628 (2012). 22101895 PMC3277358

[b18] Nakato H., Shinomiya K. & Mikawa H. Possible role of neuraminidase in the pathogenesis of arteritis and thrombocytopenia induced in rats by Erysipelothrix rhusiopathiae. Pathology, research and practice 181, 311–319, 10.1016/S0344-0338(86)80109-1 (1986). 3748879

[b19] Goswami K. & Koner B. C. Level of sialic acid residues in platelet proteins in diabetes, aging, and Hodgkin’s lymphoma: a potential role of free radicals in desialylation. Biochemical and biophysical research communications 297, 502–505 (2002). 12270122 10.1016/s0006-291x(02)02241-6

[b20] Tribulatti M. V., Mucci J., Van Rooijen N., Leguizamon M. S. & Campetella O. The trans-sialidase from Trypanosoma cruzi induces thrombocytopenia during acute Chagas’ disease by reducing the platelet sialic acid contents. Infection and immunity 73, 201–207, 10.1128/IAI.73.1.201-207.2005 (2005). 15618155 PMC538983

[b21] Rajendiran S., Lakshamanappa H. S., Zachariah B. & Nambiar S. Desialylation of plasma proteins in severe dengue infection: possible role of oxidative stress. The American journal of tropical medicine and hygiene 79, 372–377 (2008). 18784228

[b22] Josefsson E. C. . Megakaryocytes possess a functional intrinsic apoptosis pathway that must be restrained to survive and produce platelets. The Journal of experimental medicine 208, 2017–2031, 10.1084/jem.20110750 (2011). 21911424 PMC3182050

[b23] Grozovsky R. . The Ashwell-Morell receptor regulates hepatic thrombopoietin production via JAK2-STAT3 signaling. Nature medicine, 10.1038/nm.3770 (2014). PMC4303234 25485912

[b24] Bonten E., van der Spoel A., Fornerod M., Grosveld G. & d’Azzo A. Characterization of human lysosomal neuraminidase defines the molecular basis of the metabolic storage disorder sialidosis. Genes & development 10, 3156–3169 (1996). 8985184 10.1101/gad.10.24.3156

[b25] Pshezhetsky A. V. . Cloning, expression and chromosomal mapping of human lysosomal sialidase and characterization of mutations in sialidosis. Nature genetics 15, 316–320, 10.1038/ng0397-316 (1997). 9054950

[b26] Ballem P. J. . Mechanisms of thrombocytopenia in chronic autoimmune thrombocytopenic purpura. Evidence of both impaired platelet production and increased platelet clearance. The Journal of clinical investigation 80, 33–40, 10.1172/JCI113060 (1987). 3597777 PMC442198

[b27] Sorensen A. L. . Role of sialic acid for platelet life span: exposure of beta-galactose results in the rapid clearance of platelets from the circulation by asialoglycoprotein receptor-expressing liver macrophages and hepatocytes. Blood 114, 1645–1654, 10.1182/blood-2009-01-199414 (2009). 19520807 PMC2731641

[b28] Grewal P. K. The Ashwell-Morell receptor. Methods in enzymology 479, 223–241, 10.1016/S0076-6879(10)79013-3 (2010). 20816169

[b29] Li J. . Severe platelet desialylation in a patient with glycoprotein Ib/IX antibody-mediated immune thrombocytopenia and fatal pulmonary hemorrhage. Haematologica 99, e61–63, 10.3324/haematol.2013.102897 (2014). 24532041 PMC3971097

[b30] Li J. . Desialylation is a mechanism of Fc-independent platelet clearance and a therapeutic target in immune thrombocytopenia. Nature communications 6, 7737, 10.1038/ncomms8737 (2015). PMC4518313 26185093

[b31] McMillan R. The pathogenesis of chronic immune thrombocytopenic purpura. Seminars in hematology 44, S3–S11, 10.1053/j.seminhematol.2007.11.002 (2007). 18096470

[b32] Cines D. B., Bussel J. B., Liebman H. A. & Luning Prak E. T. The ITP syndrome: pathogenic and clinical diversity. Blood 113, 6511–6521, 10.1182/blood-2009-01-129155 (2009). 19395674 PMC2710913

[b33] Zhao C. . Increased cytotoxic T-lymphocyte-mediated cytotoxicity predominant in patients with idiopathic thrombocytopenic purpura without platelet autoantibodies. Haematologica 93, 1428–1430, 10.3324/haematol.12889 (2008). 18757854

[b34] Zhou H. . Interleukin 27 inhibits cytotoxic T-lymphocyte-mediated platelet destruction in primary immune thrombocytopenia. Blood 124, 3316–3319, 10.1182/blood-2014-06-580084 (2014). 25298039 PMC4239337

[b35] Santamaria P. Effector lymphocytes in autoimmunity. Current opinion in immunology 13, 663–669 (2001). 11677087 10.1016/s0952-7915(01)00276-x

[b36] Olsson B., Jernas M. & Wadenvik H. Increased plasma levels of granzymes in adult patients with chronic immune thrombocytopenia. Thrombosis and haemostasis 107, 1182–1184, 10.1160/TH12-01-0012 (2012). 22476618

[b37] Andre N. D. . Measurement of cytotoxic activity in experimental cancer. Journal of clinical laboratory analysis 18, 27–30, 10.1002/jcla.20006 (2004). 14730554 PMC6807731

[b38] Yang W. . Cytotoxic effects of T cells induced by fusion protein 6B11-pulsed dendritic cells on ovarian carcinoma cells. Gynecologic oncology 105, 238–243, 10.1016/j.ygyno.2006.04.028 (2007). 17383546

[b39] Kotze H. F. . Influence of platelet membrane sialic acid and platelet-associated IgG on ageing and sequestration of blood platelets in baboons. Thrombosis and haemostasis 70, 676–680 (1993). 8115995

[b40] Alioglu B., Tasar A., Ozen C., Selver B. & Dallar Y. An experience of oseltamivir phosphate (tamiflu) in a pediatric patient with chronic idiopathic thrombocytopenic purpura: a case report. Pathophysiology of haemostasis and thrombosis 37, 55–58, 10.1159/000321379 (2010). 21063076

[b41] Shao L. . Successful treatment with oseltamivir phosphate in a patient with chronic immune thrombocytopenia positive for anti-GPIb/IX autoantibody. Platelets 1–3, 10.3109/09537104.2014.948838 (2014). 25166956

[b42] Choi S. I., Simone J. V. & Jorney L. J. Neuraminidase-induced thrombocytopenia in rats. British journal of haematology 22, 93–101 (1972). 5060079 10.1111/j.1365-2141.1972.tb08790.x

[b43] Grottum K. A. & Jeremic M. Neuraminidase injections in rabbits. Reduced platelet surface charge, aggregation and thrombocytopenia. Thrombosis et diathesis haemorrhagica 29, 461–469 (1973). 4762260

[b44] Monti E., Preti A., Venerando B. & Borsani G. Recent development in mammalian sialidase molecular biology. Neurochemical research 27, 649–663 (2002). 12374200 10.1023/a:1020276000901

[b45] Monti E. . Sialidases in vertebrates: a family of enzymes tailored for several cell functions. Advances in carbohydrate chemistry and biochemistry 64, 403–479, 10.1016/S0065-2318(10)64007-3 (2010). 20837202

[b46] Chow L. . A murine model of severe immune thrombocytopenia is induced by antibody- and CD8+ T cell-mediated responses that are differentially sensitive to therapy. Blood 115, 1247–1253, 10.1182/blood-2009-09-244772 (2010). 20007808

[b47] Park J. H., Kim K. L. & Cho E. W. Detection of surface asialoglycoprotein receptor expression in hepatic and extra-hepatic cells using a novel monoclonal antibody. Biotechnology letters 28, 1061–1069, 10.1007/s10529-006-9064-0 (2006). 16799763

[b48] Rodeghiero F. . Standardization of terminology, definitions and outcome criteria in immune thrombocytopenic purpura of adults and children: report from an international working group. Blood 113, 2386–2393, 10.1182/blood-2008-07-162503 (2009). 19005182

[b49] Wadenvik H. & Kutti J. The *in vivo* kinetics of 111In- and 51Cr-labelled platelets: a comparative study using both stored and fresh platelets. British journal of haematology 78, 523–528 (1991). 1911343 10.1111/j.1365-2141.1991.tb04482.x

[b50] Hou M. . Mycophenolate mofetil (MMF) for the treatment of steroid-resistant idiopathic thrombocytopenic purpura. European journal of haematology 70, 353–357 (2003). 12756016 10.1034/j.1600-0609.2003.00076.x

[b51] Verhoeven A. J., Verhaar R., Gouwerok E. G. & de Korte D. The mitochondrial membrane potential in human platelets: a sensitive parameter for platelet quality. Transfusion 45, 82–89, 10.1111/j.1537-2995.2005.04023.x (2005). 15647022

[b52] Albanyan A. M., Harrison P. & Murphy M. F. Markers of platelet activation and apoptosis during storage of apheresis- and buffy coat-derived platelet concentrates for 7 days. Transfusion 49, 108–117, 10.1111/j.1537-2995.2008.01942.x (2009). 18954396

